# Cellular uptake and retention studies of silica nanoparticles utilizing senescent fibroblasts

**DOI:** 10.1038/s41598-022-26979-1

**Published:** 2023-01-10

**Authors:** Patrick M. Perrigue, Agata Henschke, Bartosz F. Grześkowiak, Łucja Przysiecka, Kaja Jaskot, Angelika Mielcarek, Emerson Coy, Sergio E. Moya

**Affiliations:** 1 NanoBioMedical Centre, Adam Mickiewicz University, Wszechnicy Piastowskiej 3, 61-614 Poznan, Poland; 2 Center for Cooperative Research in Biomaterials (CIC biomaGUNE), Basque Research and Technology Alliance (BRTA), Paseo de Miramon 182, 20014 Donostia San Sebastián, Spain

**Keywords:** Cell biology, Nanoscience and technology

## Abstract

Understanding the interplay between nanoparticles (NPs) and cells is essential to designing more efficient nanomedicines. Previous research has shown the role of the cell cycle having impact on the efficiency of cellular uptake and accumulation of NPs. However, there is a limited investigation into the biological fate of NPs in cells that are permanently withdrawn from the cell cycle. Here we utilize senescent WI-38 fibroblasts, which do not divide and provide a definitive model for tracking the biological fate of silica nanoparticles (SiNPs) independent of cell cycle. We use several methods to measure the cellular uptake kinetics and intracellular retention of SiNPs, including confocal laser scanning microscopy (CLSM), flow cytometry, and transmission electron microscopy (TEM). We demonstrate that SiNPs readily enter into senescent cells. Once internalized, SiNPs do not exit and accumulate in the cytoplasm for long term. Our study provides a basis for future development of NP-based tools that can detect and target senescent cells for therapy.

## Introduction

The use of NPs in several fields of application, such as medicine, has led to a tremendous surge in research on how nanomaterials behave inside cells. NPs used as drug carriers are constructed from a variety of substances, including lipids, metals, and natural or synthetic polymers^1^. The size, concentration, shape, material, and surface charge of NPs are augmentable parameters for enhancing their biofunctionality and tune their interaction and cellular uptake^2–5^. Improved therapeutic profiles, higher bioavailability, and lower off target toxicity of the carried therapeutic are some of the desired outcomes for NP-delivery platforms. Experimentally relevant in vitro and in vivo conditions such as cell and animal models are equally important to track the biological fate of NPs and monitor how well they function.

SiNPs exhibit high stability and low toxicity in cell applications such as drug delivery^6,7^. They readily uptake into cells via an endocytosis mechanism^8^. Once internalized, they can traffic within endosomes to arrive inside lysosomes and other cell organelles^9,10^. However, inherent cellular factors are known to influence the efficiency of these mechanisms. For example, large cells or cells in the G2/mitotic phase of the cell cycle significantly uptake more NPs^11,12^. Also, proliferation significantly reduces the amount of internalized NPs with each cell division^13^. Investigating other cellular states conducive to SiNP uptake and retention is needed and could yield new knowledge about the capacities of NP-delivery platforms.

Cellular senescence is a tumor suppression mechanism that blocks damaged cells from proliferating out of control^14^. There is ongoing research to define new markers or causes of senescence to understand its role in disease development. DNA damage, telomere shortening, mitochondrial damage, and epigenetic factors are known to activate it^15^. Senescent cells secrete various proteins and molecules that trigger inflammation linked to the development of age-related diseases^16^. The accumulation of senescent cells with organismal age was shown to reduce lifespan and be harmful to health^17,18^. The knowledge about cellular senescence being the basis of aging has prompted researchers to investigate how to remove senescent cells around the body using drug therapies^19^.

Doxorubicin-induced senescence and its associated cancer relapse have been studied in mice^20^. The results showed that non-tumor senescent cells that appear after doxorubicin treatment play an essential role in cancer relapse and the spreading to distal tissues. Eliminating senescent cells limited the manifestation of adverse reactions to chemotherapy treatment. The clinical relapse of cancer emphasizes the importance of approaching this issue more complexly at the nexus of biology and nanomedicine. There are numerous well-established nanomedical devices that circumvent off-targeting of chemotherapy^21^. From a different perspective, cellular senescence that arises in the cancer cells themselves is another challenge. A recent study demonstrates that cancer cells that acquire senescence brought on by chemotherapy can eventually re-enter the cell cycle^22^. There is even more evidence that senescence can make the surviving cancer cells after treatment more aggressive^23,24^. Several types of NPs are at the forefront to be able to deliver compounds capable of preferentially killing senescent cells^25–27^. However, for the most part, the biological fate of NPs in senescent cells is generally unknown.

In this study, we utilize senescent cells for their permanent growth arrest status to explore SiNP biological fate. We examined fluorescently labeled SiNPs traceable by CLSM and flow cytometry in senescent cells. We also used TEM to confirm that senescent cells uptake and harbor SiNPs long-term. Accordingly, senescent cells offer a different model for measuring cellular uptake kinetics and retention when compared to proliferative cells. Our research on the biological fate of NPs has the potential to be translated into better detection and targeting of a medically relevant cell type.

## Results

**Figure 1:**
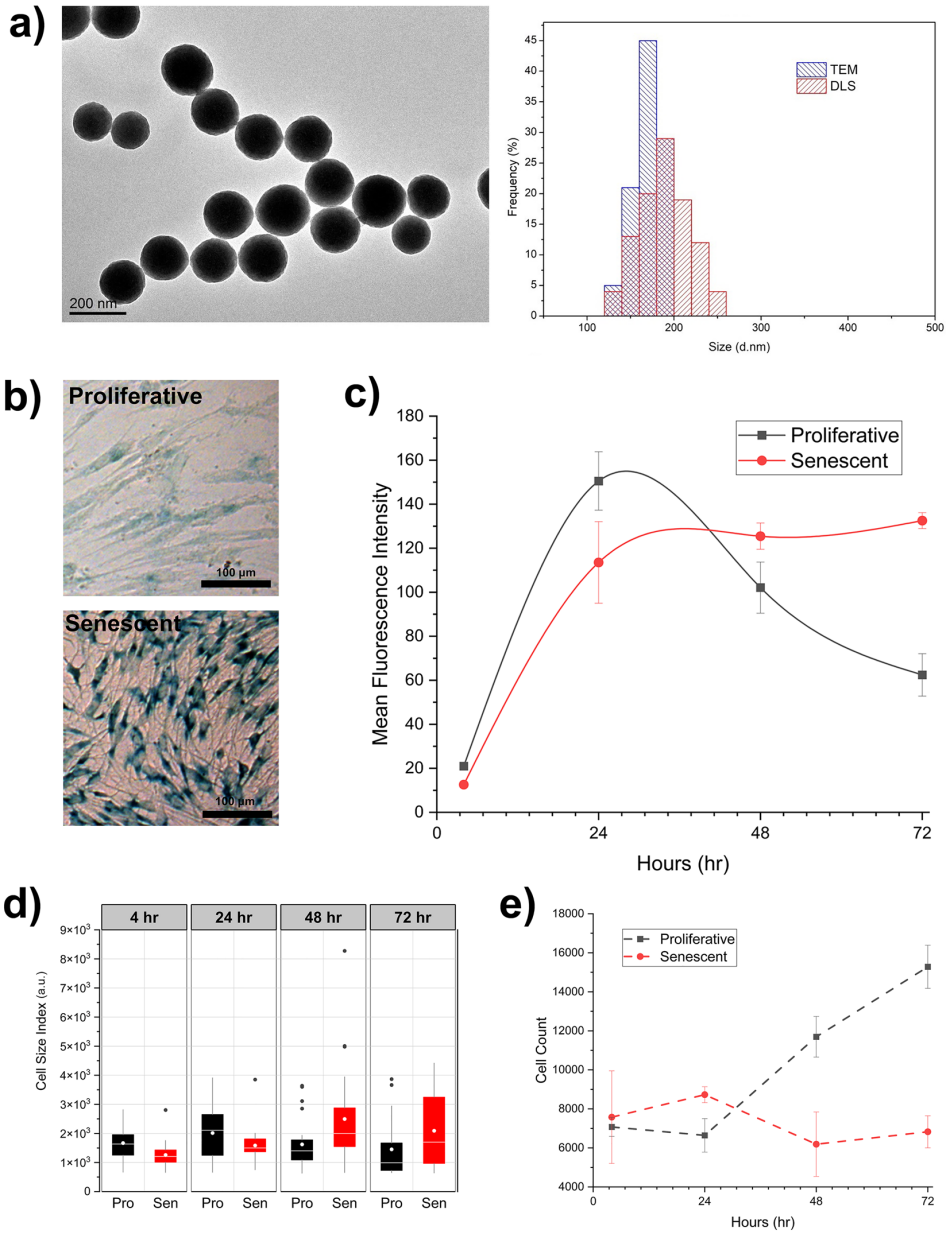
Cellular uptake kinetics of SiNPs of the 200 nm size range in proliferative and senescent WI-38 fibroblasts over a 72 h timecourse. (**a**) Electron micrographs of SiNPs. The histogram to the right shows TEM and DLS measurements. (**b**) Staining for SA-β-gal activity at 48 h to confirm cellular senescence (bluish-green stain). (**c**) Flow cytometry results for cells incubated continuously with 6.25 µg/ml of SiNPs. Error bars indicate S.E.M. (n = 3). (**d**) Box plot for cell size indexes over time (n = 25 for each condition)*.* (**e**) Graph shows the amount of cell proliferation over time. Error bars indicate S.E.M. (n = 3).

We used commercially available SiNPs with three different diameter lengths; 200, 500, and 1000 nm. TEM images of the SiNPs show they have a spherical shape and good monodispersity, with consistent diameter averages (Fig. 1a and Fig. S1a,b). Furthermore, DLS measurements provided similar size ranges, except particles of 1000 nm. Particles with the size of 1000 nm were aggregating together showing an increased DLS hydrodynamic diameter of approximately 5000 nm. Polydispersity of these particles was also higher (0.8 ± 0.1), compared to NPs with size of 200 nm and 500 nm (0.04 ± 0.01, 0.03 ± 0.01, respectively). All NPs were negatively charged and displayed a ζ-potential of − 50 ± 3 mV.

Next, we established cellular conditions to investigate SiNP biological fate: proliferative (dividing) and senescent cells. We derived senescent cells from WI-38 human lung embryonic fibroblast cell line^28,29^. For the induction of senescence, we utilized a previously described method^30^. We quickly validated the cells for our experiments using a staining protocol for senescence-associated beta-galactosidase (SA-βgal) activity, a characteristic biomarker of WI-38 fibroblasts undergoing senescence^31^. The results show a robust increase for SA-β-gal+ after the treatment, indicating that cells acquire a senescence phenotype (Fig. 1b). The proliferative cells, which served as the control, were WI-38 fibroblasts under standard culturing conditions.

Next, we studied the cellular uptake kinetics of SiNPs in proliferative and senescent cells. The cell types were plated in parallel, incubated with SiNPs continuously without changing the cell media, and analyzed by flow cytometry at time points over a 72 h period. We found that the uptake patterns for 200, 500, and 1000 nm SiNPs were different in proliferative versus senescent cells (Fig. 1c and Fig. S2a,b). Simultaneously using flow cytometry, we could monitor cell size and proliferation to explain the observable differences in the uptake patterns for each condition. We noticed that the senescent cells at the start of the experiment had uniformly small cell sizes that eventually grew larger with time (Fig. 1d). In contrast, the overall population of proliferative cells enter the G2/M phase of the cell cycle which produces larger cell sizes on average and increases their propensity for NP uptake^12^. These differences in cell sizes explain why proliferative cells had more SiNP content than the senescent cells at the initial stage of the time course between 4 and 24 h. Cell proliferation also had an influence in these experiments (Fig. 1e). Counting the cells each day by flow cytometry shows proliferative cells experienced an initial 48 h lag phase before eventually dividing and diluting the SiNPs inside them. By contrast, there was no increase in the cell counts for senescent cells. This suggests that both cell types could uptake SiNPs, but senescent cells were unable dilute them. Altogether, our data suggest cell size and proliferation contribute to the difference in cell uptake between proliferative and senescent cells.

**Figure 2:**
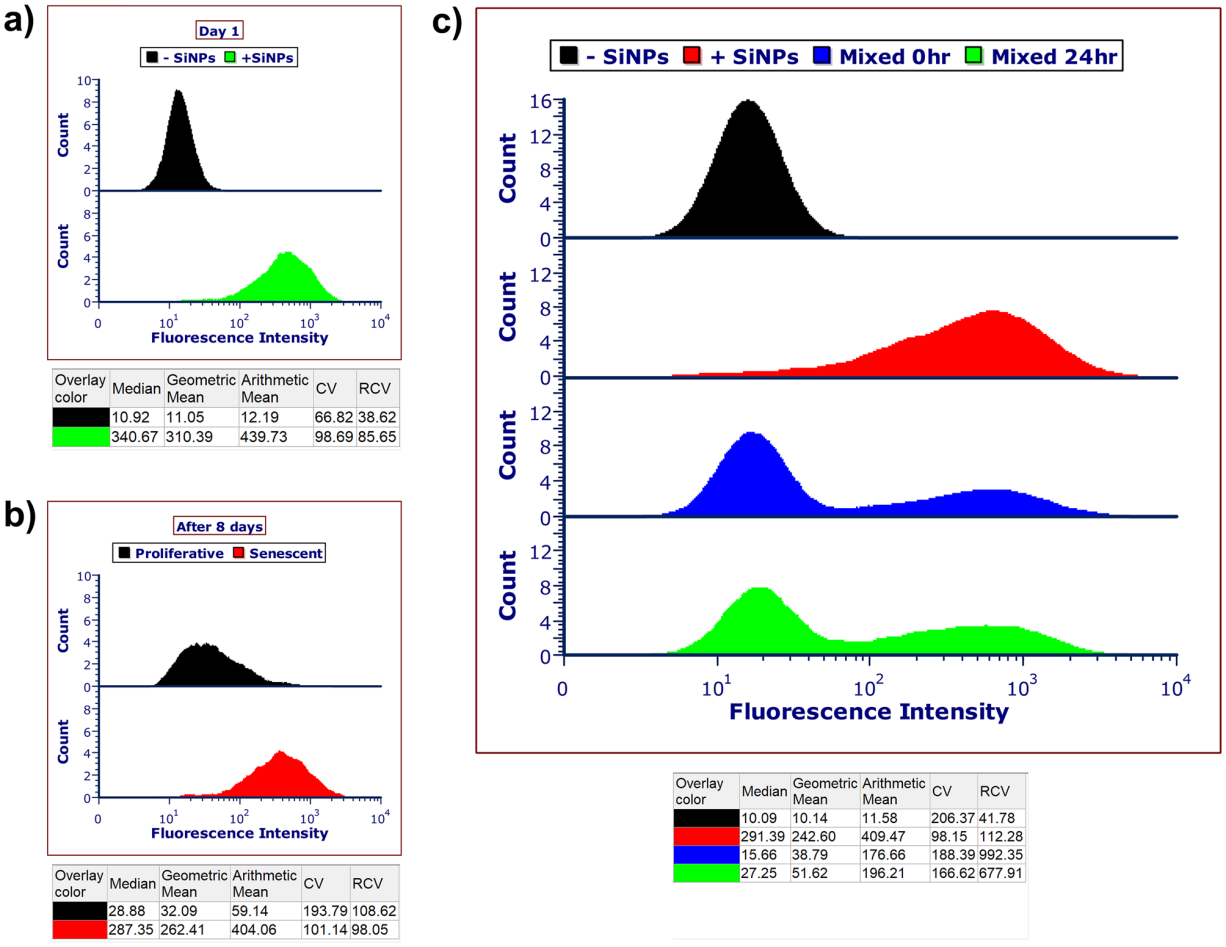
SiNP retention inside senescent cells and transfer between senescent cells is negligible. (**a**) Proliferative WI-38 fibroblasts were incubated with 25 μg/ml SiNPs for 80 min. On day 1, the cells with SiNPs (+SiNPs) were analyzed by flow cytometry alongside control cells with no silica NPs (−SiNPs). (**b**) The +SiNPs cells from (**a**) were split into two treatment protocols for continued growth and senescence induction (see “Methods” section for details). After 8 days in culture, flow cytometry revealed senescent cells had retained their SiNPs while proliferative cells diluted their content with each cell division. (**c**) Representative flow cytometry histogram showing the fluorescence intensity of +SiNPs senescent cells after 10 days compared to −SiNPs control senescent cells without. The +SiNPs and −SiNPs populations were mixed and analyzed by flow cytometry at 0 h and 24 h. The +SiNP population between 0 to 24 h increased by 8.6 ± 4.4% (n = 3).

To support the idea that SiNPs persist inside for the long term, we derived senescent cells from proliferative WI-38 fibroblasts that already contained SiNPs and analyzed them after 8 days in culture. Notably, by first loading the SiNPs into cells and then inducing senescence, we achieved the same starting material for senescent and a proliferative control population at the commencement of the experiment. Using flow cytometry, we show that senescent cells after 8 days have a similar fluorescence profile to the proliferative control cells on day 1 (Fig. 2a,b). In contrast, the proliferative cells diluted their content with each cell division, indicated by their decreased fluorescence signal. Experiments were carried out for 500 nm and 1000 nm SiNPs and yielded comparable results (Fig. S3a,b). This data suggests that the SiNPs persist inside senescent cells.

Another experiment assessed the possibility of SiNP transfer between cells. We briefly incubated senescent cells with SiNPs, washed out the leftover, and maintained the cell culture with normal passaging and media changes. After confirming these cells remained fluorescent for 10 days, we combined them with normal senescent cells at a 1:1 ratio (Fig. 2c). Flow cytometry analysis of the co-culture before and after 24 h exhibited a bimodal distribution for fluorescence indicative of two distinct populations of cells. The number of senescent cells showing positivity for fluorescence after 24 h increased by only 8.6 ± 4.4%, suggesting the transfer of SiNPs between the cell populations was low.

**Figure 3:**
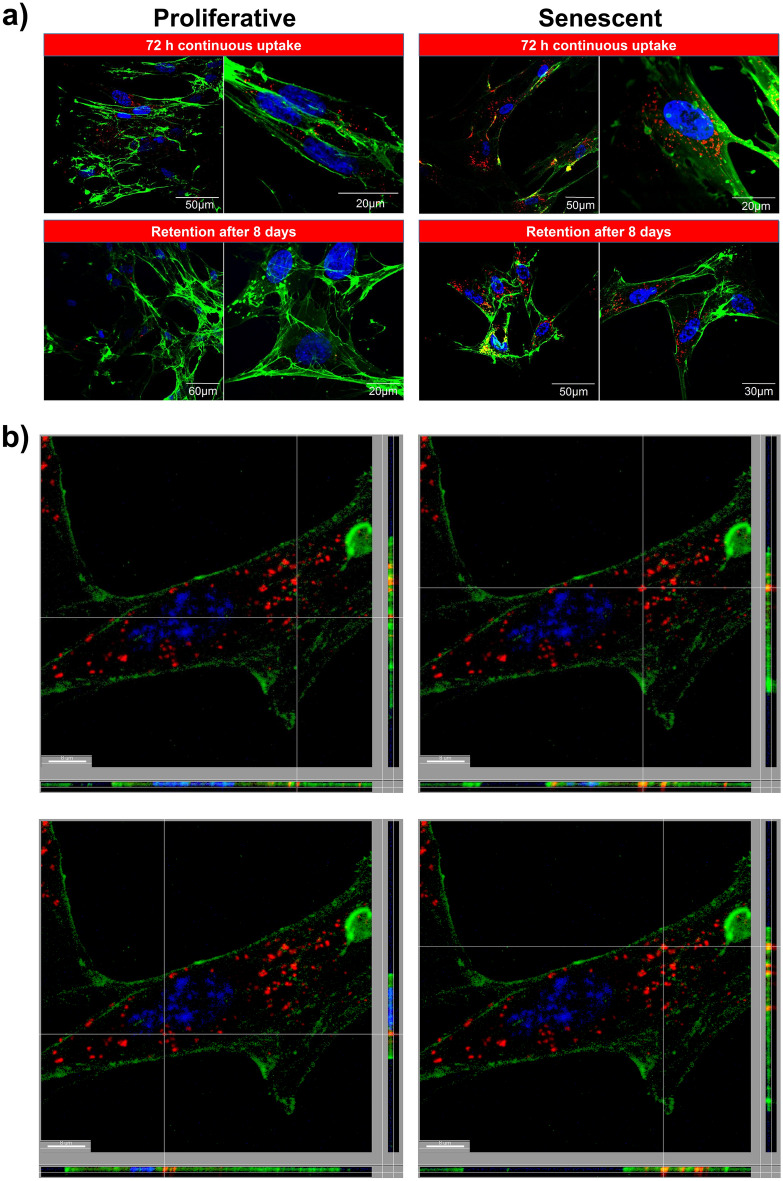
In depth analysis for the uptake and retention of 200 nm SiNPs. (**a**) CLSM images showing the comparison of proliferative and senescent cells with 72 h of continuous uptake and retention after 8 days. (**b**) Z-stack slice of senescent cell with SiNP retention after 8 days. The images show four different focus points within the same cell. The region of interest on each image is set where the lines intersect and the x–z and y–z axis are shown along the side of each x–y slice. (**a**, **b**) Staining with Hoechst 33342 (blue) and conconavalin A (green) shows the nucleus and cell membranes, respectively, in relation to SiNPs (red).

**Figure 4:**
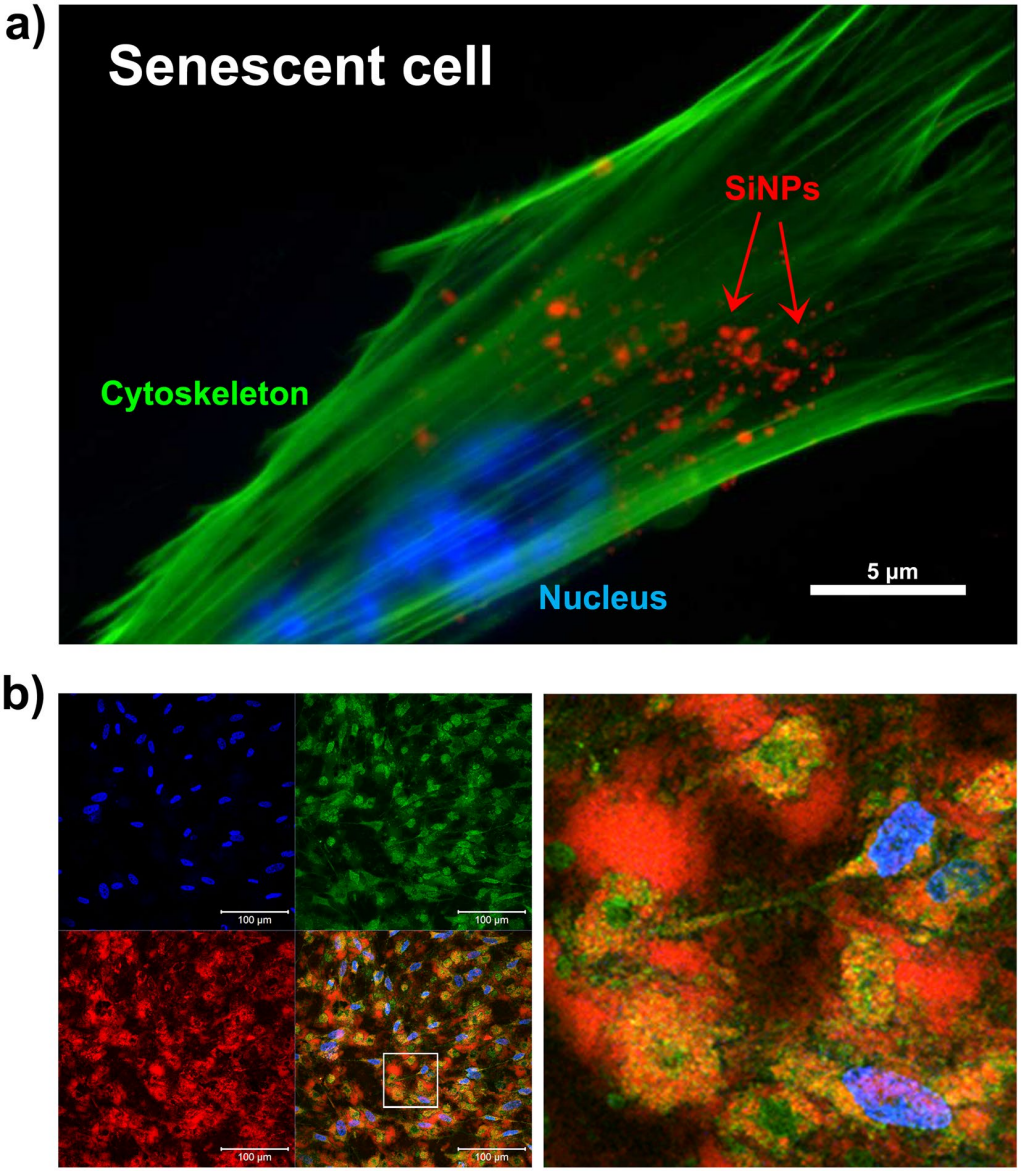
Intracellular accumulation of 200 nm SiNPs. (**a**) Senescent cells were incubated with 25 µg/ml of SiNPs for 80 min and imaged at 24 h using a fluorescence microscope. Staining with Hoechst 33342 (blue) and phalloidin-488 (green) shows the nucleus and cytoskeleton, respectively, in relation to internalized SiNPs (red). (**b**) Senescent cells were incubated with 166.66 µg/ml for 24 h and imaged by CLSM. Staining with Hoechst 33342 (blue) and conconavalin A (green) shows the nucleus and cell membranes, respectively, in relation to SiNPs (red). The right panel is a magnified region from the square area marked in the left panel.

To confirm the internalization of 200, 500 and 1000 SiNPs we performed CLSM on cells from the previous uptake and retention experiments. As expected, the samples prepared for assessing retention after 8 days had significantly less SiNPs at the plasma membrane compared to cells after 72 h continuous uptake (Fig. 3a and Fig. S4a). The images also show that there was significant dilution of SiNPs in proliferative cells in contrast to senescent cells. Due to the low amount of SiNPs in proliferative cells we performed additional image analysis for the senescent cells including confocal z-stack slice reconstructions and 3D-images showing SiNPs in the cytoplasm (Fig. 3b, Fig. S4b, and Supplemental Videos 1–3). Additional staining of F-actin also confirmed this, which shows the fluorescence signal for SiNPs overlays within the cytoskeleton of senescent cells (Fig. 4a and Fig. S5a,b). To further support that SiNPs accumulate in senescent cells, a higher concentration of SiNPs (166.66 μg/ml) was used to simulate overloading of senescent cells. Notably, this concentration was reported to be cytotoxic in other studies^32,33^. CLSM images show the release of SiNPs from senescent cells due to plasma membrane leakage (Fig. 4b). The result suggests a critical capacity for carrying SiNPs related to cytotoxicity.

**Figure 5:**
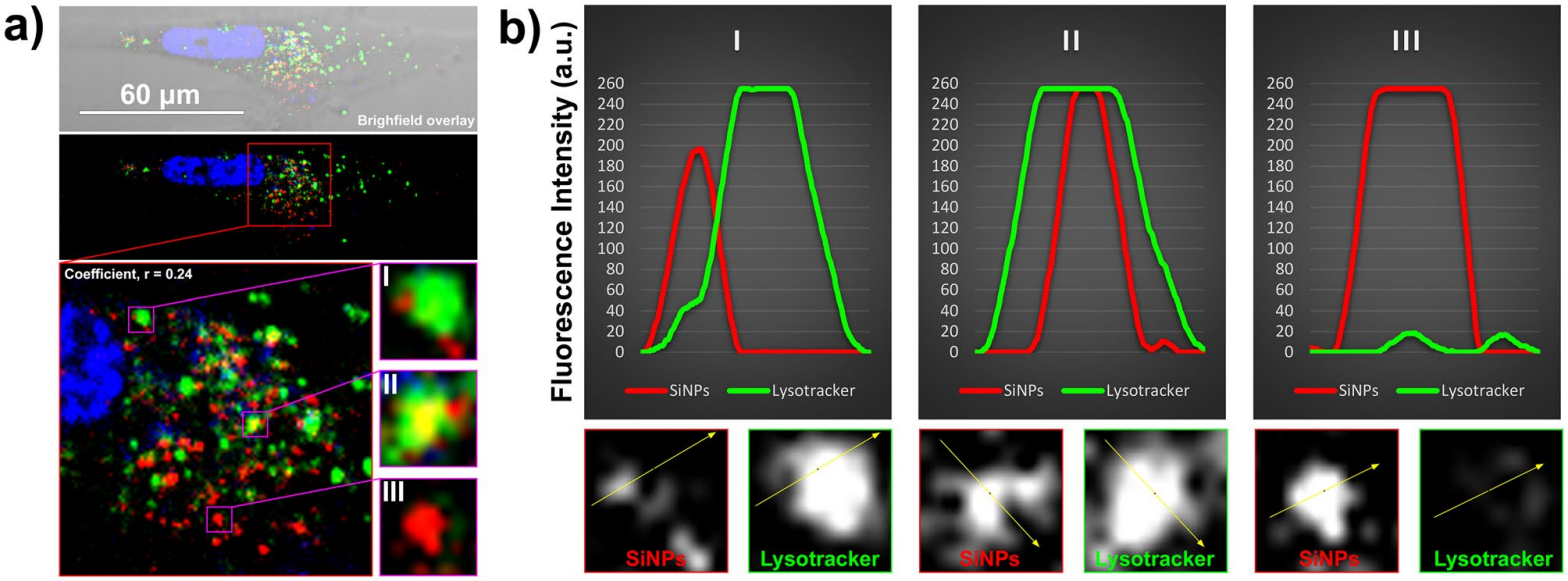
Co-localization of 200 nm SiNPs with lysotracker inside senescent cells. (**a**) Live imaging of senescent cells using CLSM. Staining with Hoechst 33342 (blue) and lysotracker (green) shows the nucleus and acidic cell compartments, respectively, in the same confocal plane as SiNPs (red). Colocalization analysis found overlap between lysotracker and SiNPs for the cell in the image with a Pearson’s correlation coefficient, r = 0.24. SiNPs and their relationship to lysotracker are denoted as I, II, and III and described further in the main text. (**b**) Quantification of the fluorescence images shown in (**a**) using a line analysis. The fluorescence channels were first split using Image J software and transformed to binary formats. The signals along the yellow line drawn for examples I, II, and III were plotted as overlaid histograms.

Next, we used lysotracker dye followed by CLSM to confirm the subcellular localization of 200 nm SiNPs. Senescent cells displayed fluorescence for both SiNPs and lysotracker dye in the same confocal plane (Fig. 5a). Analysis of the fluorescence signals shows a correlation overlap between SiNPs and low pH compartments (Coefficient, r = 0.24). Zooming into regions of the confocal image revealed the SiNPs that do not perfectly overlap with low pH compartments are nevertheless nearby lysotracker signal. We observed three types of SiNP associations to lysotracker signal denoted as, I (adjunct to lysotracker), II (overlap with lysotracker) and III (not associated with lysotracker). A line analysis over these fluorescence signals quantified the relationships more closely (Fig. 5b). These locations for SiNPs implicate the endo-lysosomal pathway in the mechanism for longterm retention.

**Figure 6:**
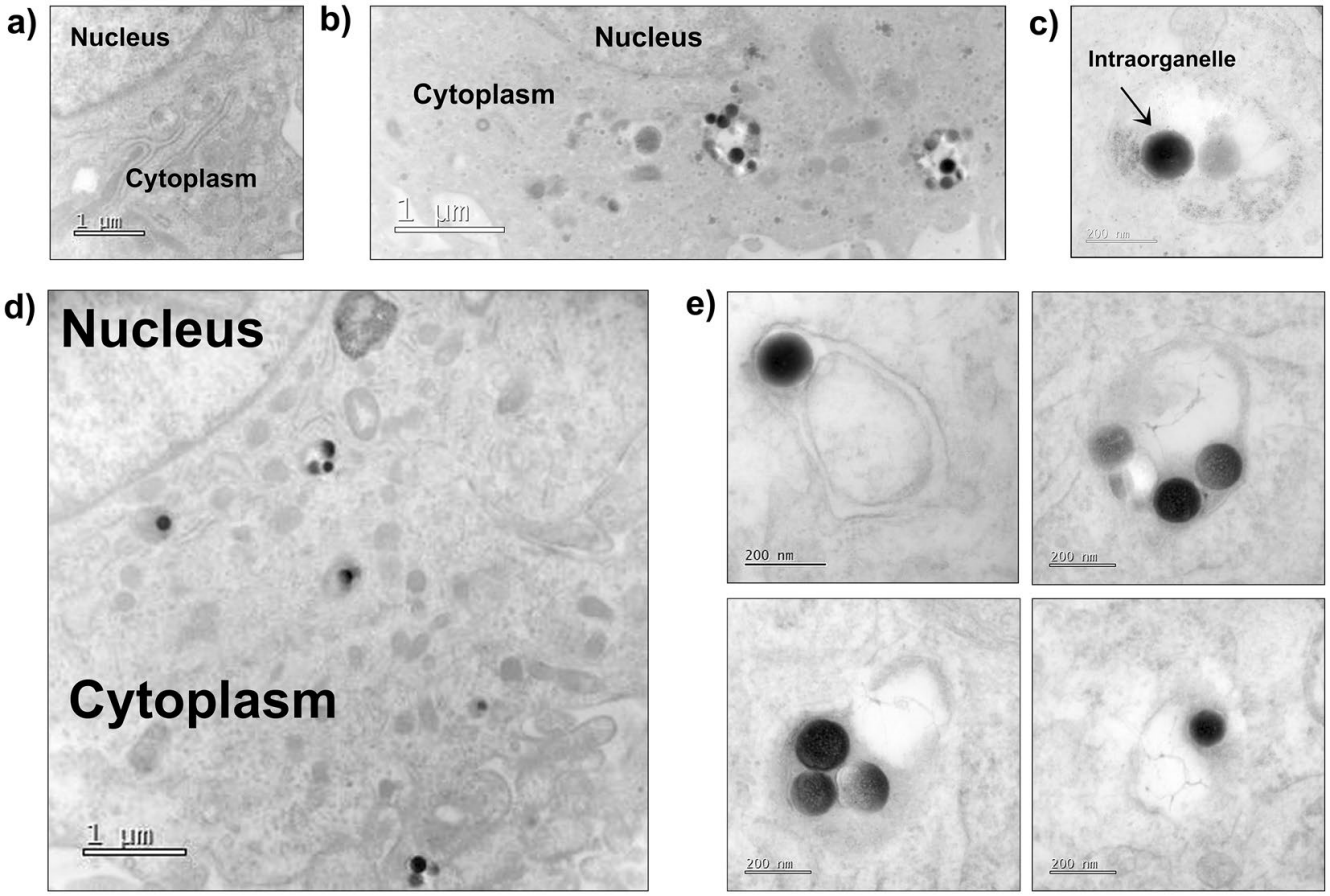
Permeant growth arrest allows for long term biological fate inside senescent cells. (**a**) Electron micrographs of senescent cells without SiNPs (control), (**b**) with SiNPs in the cytoplasm, and (**c**) SiNPs inside an organelle at 24 h. (**d**) Electron micrographs of a senescent cell with SiNPs in the cytoplasm, and (**e**) SiNPs intercalated into the membranes of organelles at 12 days.

We also inspected the senescent cells by TEM in case rhodamine became uncoupled from SiNPs over the more extended culturing periods. SiNPs were found inside organelles resembling endosomes at 24 h (Fig. 6a–c). More images after 12 days, show SiNPs still in the cytoplasm with some intercalated into the lipid membrane of organelles resembling lysosomes (Fig. 6d,e). These data demonstrate that senescent cells definitively internalize and retain SiNPs versus the possibility of being exclusively stuck on the plasma membrane.

## Discussion

There are several advantages of using permanently growth arrested (senescent) cells to investigate the biological fate of SiNPs. First, they represent a cell status in the aging human body. Second, senescent cells have pronounced morphological features for pinpointing the subcellular location of NPs. Third, there is no dilution of NPs from cell division. The majority of studies which uncovered the mechanisms of SiNPs were elucidated on normally dividing and cancer cell lines. Reassessing the functions of SiNPs in senescent cells is relevant because it has the potential to uncover new applications. For example, fluorescent SiNPs are helpful for long-term cell tracking studies^34^. We not only reconfirm this, but demonstrate SiNPs are also suitable for monitoring the long-term growth arrest of senescent cells.

Flow cytometry is an established method to check for the cellular uptake of NPs^35^. Using flow cytometry, we investigated the uptake kinetics for proliferative and senescent cells in a side-by-side comparison for three different sizes of SiNPs. A critical issue for measuring uptake on proliferative cells is that it requires a window of minutes or hours, so that cell division has a negligible impact. Our experiments clearly show the population doubling-time of WI-38 fibroblasts impeded upon the cellular uptake of SiNPs. In contrast, we observed uptake of SiNPs in senescent cells over several days opening the possibility of future studies on the long-term biological responses to nanomaterials.

We measured the uptake of SiNPs using a concentration below the cytotoxicity threshold. However, when senescent cells become overloaded with SiNPs, they have plasma membrane leakage. The general mechanism explains why SiNPs might be more cytotoxic to certain cells. Our results are in line with idea that lysosomal leakage caused by SiNPs can manifest as cytotoxicity^36,37^. We can compare another study that investigated the increased sensitivity of senescent cells to quantum dots^38^. In that study, the authors observed cell death from lysosome leakage of quantum dots occurs to a lesser degree in young proliferative cells. Several other studies highlight that cytotoxicity of SiNPs is concentration- and cell type-dependent^39–41^. This further supports the idea that metabolic activity and ability for cells to dilute foreign material is crucial for survival.

We demonstrated proliferative and senescent WI-38 fibroblasts readily uptake SiNPs, however, senescent cells do no dilute their SiNPs. Furthermore, the transfer of SiNPs between senescent cells was minimal. In our study we overcame several issues with analyzing the retention and exocytosis of SiNPs. Senescent cells inherently resist apoptosis and are metabolically active^42^ and therefore the chance of dying cells releasing their content into the media was minimal. There are conflicting reports about the retention and exocytosis of SiNPs which may be partially explained by differences in NP-size, the cell types and/or the experimental setups used to monitor it^43^. SiNP retention in senescent cells is in line with a recent study that shows exocytosis of NPs is negligable^13^. In another report, Slowing et al. showed that the exocytosis of mesoporous SiNPs differs between healthy cells with minimal particle transfer compared to malignant cells with a large amount of particle transfer^44^.

In our experimental conditions, the washes, media exchanges, and passaging of the cells over the timeframe of several days help to remove the leftover residual SiNPs not inside cells. Because proliferative cells had significantly less SiNPs due to dilution we focused our effort on to studying the locations of SiNPs in senescent cells. We visualized the internalization of 200 nm SiNPs in the most detail by confocal microscopy and TEM. The study of colocalization with lysotracker shows some SiNPs intercalated into the membranes of lysosomes. One limitation of our study was we did not track the percentage location of every particle and whether the endo-lysosomal pathway occupancy we observed was permanent or transient. Despite all the evidence that SiNPs internalize, our analysis may not account for some SiNPs presented onto the outer plasma membrane. Therefore, future directions should include a complete investigation by single particle tracking in living cells and at a higher time-resolution.

The removal of senescent cells represents a paradigm shift in medicine^45^. Targeted selective killing of senescent cells using platforms based on nanomaterials has the potential to fight various age-related diseases^46–50^. A fundamental issue to advance on the application of nanomedicine tools onto aging is to understand how NPs uptake and accumulate under senescence conditions. Our findings identify the retention kinetics of NPs as a potential exploit to circumvent their off-targeting in proliferative cells while restricting drug toxicity to senescent cells where it is desirable. Senescent WI-38 fibroblasts provide a definitive model tool for the purposes of tracking SiNPs. Once internalized, SiNPs do not exit senescent cells, do not degrade, and reside inside endosomes and lysosomes, which is their biological fate. The results and methodology here presented are not only important for senescent cells studies, but could be pivotal on the study of fate and long term NPs stability by in in-situ experiments. Finally, further studies are needed, especially those aimed to determine the fate of composite, polymeric and degradable nanoprobes for biomedical applications.

## Methods

### Characterization techniques

Fluorescent SiNPs with excitation/emission of 569/585nm were purchased from Kisker Biotech GmbH & Co. KG. (K version: PSI-R02, -R05, -R1.0, Steinfurt, Germany). Rhodamine B was covalently attached to alkoxysilanes and incorporated in the silica matrix of the particles. The particle diameter of SiNPs was evaluated by Dynamic Light Scattering (DLS) on a Zetasizer Nano ZS (Malvern Panalytical). NPs (0.1 mg/ml) were dispersed at room temperature in aqueous solutions by using an ultrasound bath for 10 min. Their surface charge was also measured by recording ζ-potential with the Zetasizer Nano-ZS. TEM images of SiNPs were recorded on Transmission Electron Microscope Jeol 1400, operation at 120 kV, and on High Resolution Transmission Electron Microscope (HRTEM) Jeol ARM 200F, operation at 200 kV.

### Cell culture

We obtained the WI-38 (CCL-75) cell line from American Type Culture Collection (Manassas, Virginia, USA). Cells were cultured in Eagle’s minimum essential medium (EMEM) (M4655, Sigma St. Louis, MO, USA), USA), with the following additives, sodium pyruvate (Sigma S8636, St. Louis, MO, USA), 10% fetal bovine serum (EURx E5050-02, Gdansk, Poland) and antibiotic/antimycotic solution, at 1× concentrations (Sigma A5955, St. Louis, MO, USA). The media were filtered using a 0.22-micron PES disc syringe filter (Millipore; Cat No. S2GPU05RE, Burlington, MA, USA). All cell cultures were incubated in a humidified chamber containing 5% CO_2_ at 37 °C. For the culturing conditions and the induction of senescence, we used the following described methods and reagents^30^. Briefly, B-27 supplement (50×) (ThermoFisher Scientific; Cat. No. 17504044, Waltham, MA, USA), fibroblast growth factor; FGF (Sigma-Aldrich; F0291, St. Louis, MO, USA), epidermal growth factor; EGF (Sigma-Aldrich; E9644) were prepared for cell culture at a stock concentration of 50 µg/ml. The solvent for growth factor resuspension was a 1% BSA solution in PBS, that was filtered using a 0.22-micron PES syringe disc filter (Millipore; Cat. No. SLGP033RS). The senescence induction was carried out on sub-confluent cell cultures in a T-25 flask by adding 60 µl of B-27 (50×), 3 µl of EGF and 3 µl of FGF stock solutions directly to the 3 ml of culture medium already in the T-25 flask. The treatment lasted 5 days with a complete change of the basal culture medium occurring on the third day. To maintain senescence for long-term experiments, we treated cells for 2 days, passaged them, and treated a second time for 3 more days to limit the outgrowth of any that were non-senescent.

### Flow cytometry

We performed cellular uptake kinetics in 12-well plates (Biologix Europe GmbH; 07-6012). For the preparation of the experiment, we first added 500 µl of cell culture media containing a 2× desired concentration to each well. Next, 500 µl of culture media containing 10,000 cells were added on top of the volume inside the wells to achieve a final concentration of 1× SiNPs in the total volume of 1 ml. Importantly, each time point had its 12-well plate, so that sample harvesting of early time points did not disturb the later time points.

Experiments for measuring cellular retention were setup using a 70% confluent T-75 flask of proliferative WI-38 fibroblasts with 25 µg/ml of SiNPs for 80 min. The cell cultures were washed three times with HBSS (Hanks buffered salt solution; GIBCO) to remove remaining SiNPs. The day after, the culture was split into two T-25 flasks for senescence induction or continued proliferation. The cells received a media change on day 3, and on the day 5, the proliferative and senescent cells were both passaged at a ratio of 1:2. Afterwards the cell cultures were grown in media containing FBS until analyzed by flow cytometry on day 8.

Each sample for flow cytometry analysis was harvested by first removing the culture medium, washing three times with HBSS, and then incubation with trypsin solution at 37 °C. After a few minutes the trypsin was inactivated by adding an equal or greater volume of cell culture medium. A portion of the volume (trypsin + media) was transferred to a 1.5 ml Eppendorf tube and centrifuged at 1200 rpm to pellet the cells. Afterwards the cell pellet was suspended in 150–200 µl of 1 × Muse Assay buffer with light pipetting and vortexing. All samples were kept on ice while waiting for analysis. Flow cytometry was performed using a Muse Cell Analyzer (Luminex Corporation). We adapted the machine settings for RFP-LC3 Reporter Autophagy Assay to be able to detect rhodamine. The gating parameters were set according to cell size and LC3-RFP fluorescence channel. The flow cytometer was setup to collect 500–2000 events per sample. Data for mean fluorescence intensity, cell size index, and cell counts were collected from the flow cytometer and plotted in a graph using OriginLab Software (OriginLab Corporation, Northampton, MA, USA). Normalization of the data, stats, and histograms for the retention experiments were performed using FCS Express 7 cytometry software (De Novo software, Pasadena, CA, USA).

### Cell staining, fluorescence microscopy, and CLSM

Senescent cells were plated on a Lab-Tek™ Flask on Slide (Thermo Scientific; 170920). The Senescence Cells Histochemical Staining Kit (Sigma; CS0030-1KT) was used in accordance with the manufacturer’s protocol to detect SA-β-gal activity. For the immunostaining, fixation of the cells in 4% formaldehyde, followed by permeabilization with 0.1% Triton X-100 solution and blocking with 5% BSA. However, the permeabilization step was omitted when staining for cell membranes. The F-actin were stained with Phalloidin-Atto 488 (Sigma-Aldrich; 49409) and membranes with Concanavalin A-Alexa 488 conjugate (Invitrogen; C11252). LysoTracker™ Green DND-26 (Invitrogen; L7526) was incubated on cells for a total of 2 h in accordance with the manufacturer’s protocol. Hoechst 33342 (Invitrogen; H3570) stained the nuclei of living or fixed cells. Some images were taken using a Zeiss Axio Imager M2 microscope as indicated in the text. For more in depth visualization, cells were imaged using a confocal laser scanning microscope (Olympus FV1000, Japan). Image acquisition was performed with a 60× objective (1.4 oil immersion lens) and analyzed using FV10-ASW software (Olympus). Images of the SiNPs were taken using 559 nm excitation and 575–625 nm emission filters. The fluorescence from cell membranes was visualized using 488 nm excitation and 495–545 nm emission filters, whereas nuclei fluorescence was detected using 405 nm excitation source and 425–475 nm emission filters. The 3D-scan of the samples were performed using Z-stack mode measurements and analyzed with Imaris software (Bitplane). Image analyses was performed using Colocalization Finder plugin and the analyze plot profile function in ImageJ software.

### Transmission electron microscopy

The cellular uptake and distribution of SiNPs in senescent cells was analyzed by TEM according to Schrand et al.^51^ procedure with minor modification. Briefly, senescent WI-38 fibroblasts were treated with 25 µg/ml of SiNPs for 80 min, washed three times with HBSS to remove remaining SiNPs and maintained T-25 flask regularly after passaging. The cells for analysis were trypsinized and then centrifuged at 1200 rpm for 5 min, followed by fixation with 4% formaldehyde and 1% glutaraldehyde in 0.1 M PBS (pH 7.4) solution for 2 h. After fixation, the samples were stored in 8% (0.2 M) sucrose in PBS. The samples were then postfixed in 1% osmium tetroxide for 1 h at room temperature, dehydrated through a graded series of ethanol concentrations (50, 70, 80, 90, 96, and 100%) and embedded in epoxy resin. Ultrathin sections were prepared using ultramicrotome (RMC PowerTome PT-XL), collected onto TEM grids, stained with 1% uranyl acetate. Images were taken using a HRTEM Jeol ARM 200F.

## Supplementary Information

Supplementary Information.

Supplementary Video 1.

## Data Availability

The data that support the findings of this study are available from the corresponding author, P. M. P., upon reasonable request.
