# Supplementary material for: Cellular uptake and retention studies of silica nanoparticles utilizing senescent fibroblasts
Source: Sci Rep. 2023 Jan 10;13:475. doi: 10.1038/s41598-022-26979-1 (PMC9832065; doi:10.1038/s41598-022-26979-1)
Supplement: Supplementary file 1 — Supplementary Information. [file 41598_2022_26979_MOESM1_ESM.docx]

**Cellular Uptake and Retention Studies of Silica Nanoparticles Utilizing Senescent Fibroblasts**

Patrick M. Perrigue^a*^, Agata Henschke^a^, Bartosz F. Grześkowiak^a^, Łucja Przysiecka^a^, Kaja Jaskot^a^, Angelika Mielcarek^a^, Emerson Coy^a*^, Sergio E. Moya^b*^

^a^NanoBioMedical Centre, Adam Mickiewicz University, Wszechnicy Piastowskiej 3, 61-614 Poznan, Poland

^b^Center for Cooperative Research in Biomaterials (CIC biomaGUNE), Basque Research and Technology Alliance (BRTA), Paseo de Miramon 182, 20014 Donostia San Sebastián, Spain

***Corresponding authors**

**P. M. P.** [**patrick.perrigue@amu.edu.pl**](mailto:patrick.perrigue@amu.edu.pl)

**E.C.** [**coyeme@amu.edu.pl**](mailto:coyeme@amu.edu.pl)

**S.E.M.** [**smoya@cicbiomagune.es**](mailto:smoya@cicbiomagune.es)

**Table of Contents**

**Page**

**Figure S1.** Physical characterization of 500 and 1000 nm SiNPs **S-2.**

**Figure S2.** Flow cytometry for uptake of 500 nm and 1000 nm  **S-3.**

**Figure S3.** Flow cytometry for retention of 500 nm and 1000 nm **S-4.**

**Figure S4.** CLSM of cells incubated with 500 nm and 1000 nm **S-5.**

**Figure S5.** Cytoskeleton staining of cells with 500 nm and 1000 nm **S-6.**

**Supplementary Video 1** 3D image reconstruction of 200 nm at 8 days

**Supplementary Video 2** 3D image reconstruction of 500 nm at 8 days

**Supplementary Video 3** 3D image reconstruction of 1000 nm at 8 days

**Figure S1. a)** Electron micrographs of SiNPs with different sizes including, 500 nm and **b)** 1000 nm. Histogram to the right in **a)** shows TEM and DLS measurements of SiNPs of the 500 nm size range.


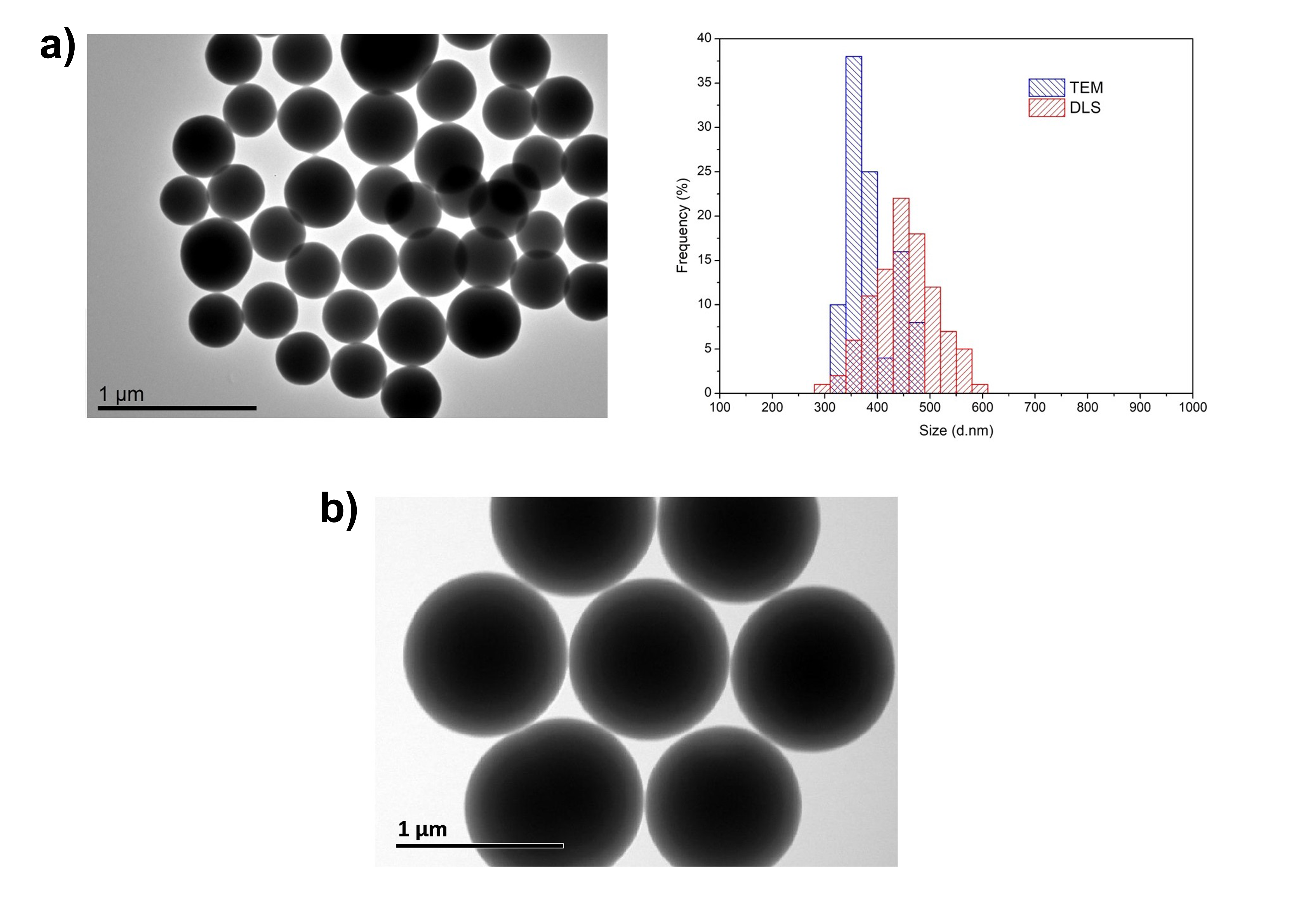

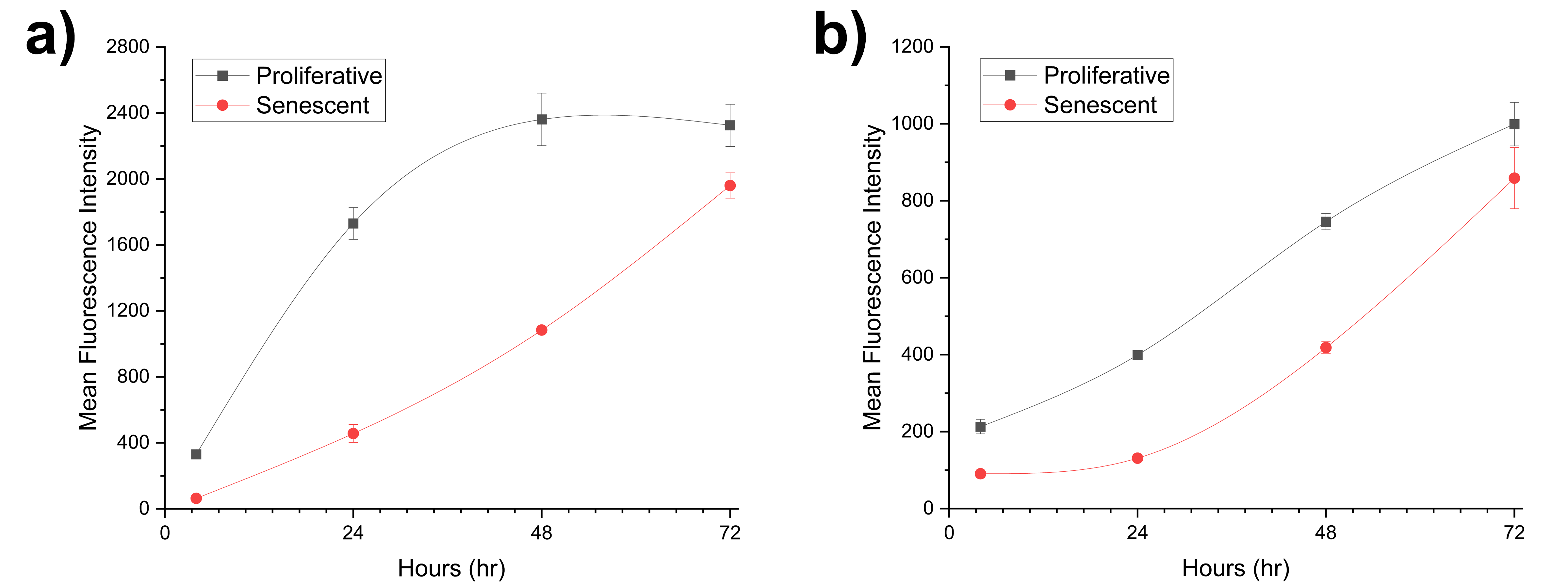


**Figure S2.** Flow cytometry results for cells incubated continuously with **a)** 500 and **b)** 1000 nm SiNPs. The concentration of SiNPs was 6.25 µg/ml. Error bars indicate s.e.m. (n = 3).

**
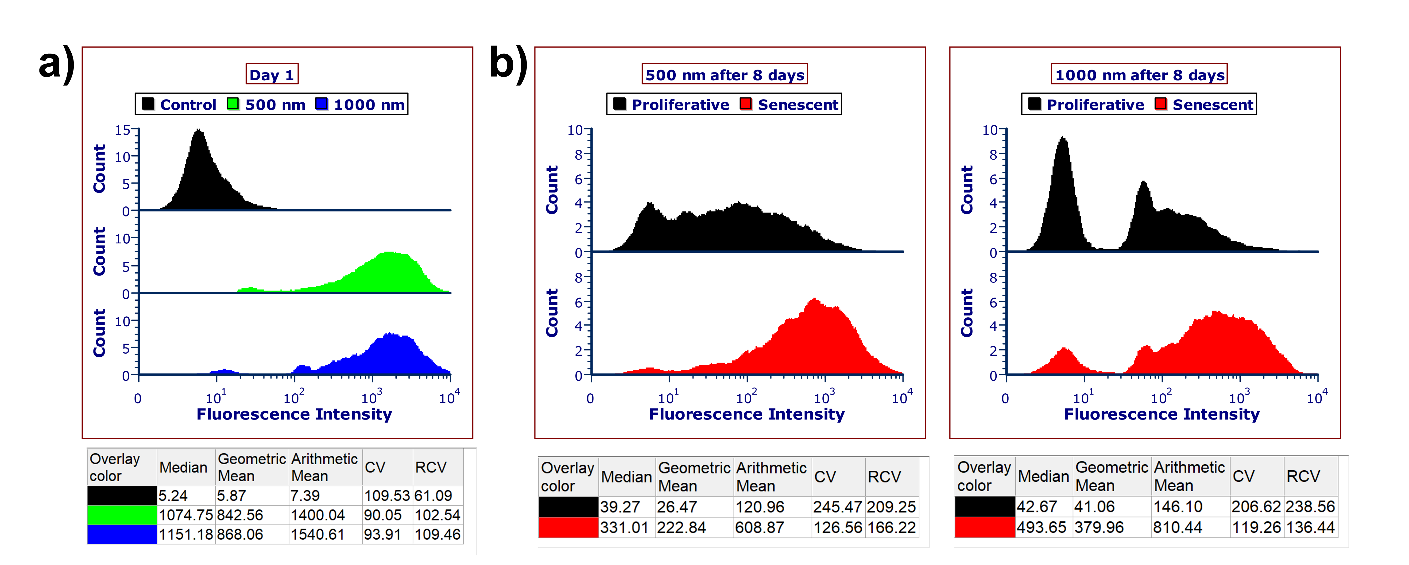
**

**Figure S3. Flow cytometry showing retention of 500 nm and 1000 nm SiNPs inside senescent cells. a)** Proliferative WI-38 fibroblasts were loaded with 500 nm and 1000 nm SiNPs. The histograms show the amount of fluorescence at day 1 following their brief incubation with SiNPs and before splitting into two treatment conditions; proliferative versus senescent. **b)** Histograms show the subsequent fluorescence profiles for proliferative and senescent cells after 8 days in culture.


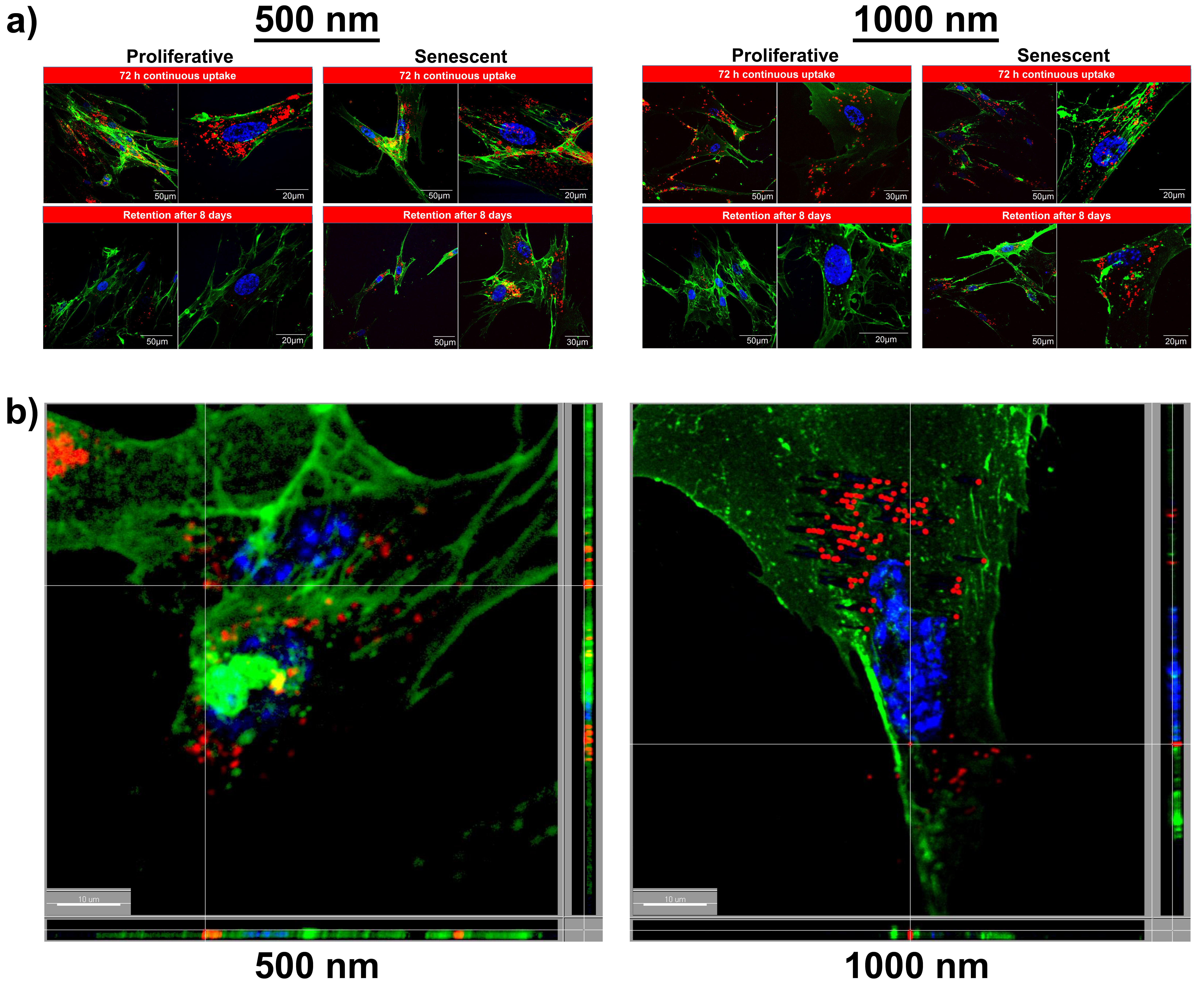


**Figure S4.** Staining with Hoechst 33342 (blue) and conconavalin A (green) shows the nucleus and cell membranes, respectively, in relation to SiNPs (red). **a)** CLSM images showing the comparison of proliferative and senescent cells with 72 h of continuous uptake and retention after 8 days. **b)** Z-stack slices of senescent cells with SiNP retention after 8 days. The region of interest on each image is set where the lines intersect and the x-z and y-z axis are shown along the side of each x-y slice. SiNP sizes of 500 and 1000 nm are indicated in the panels.


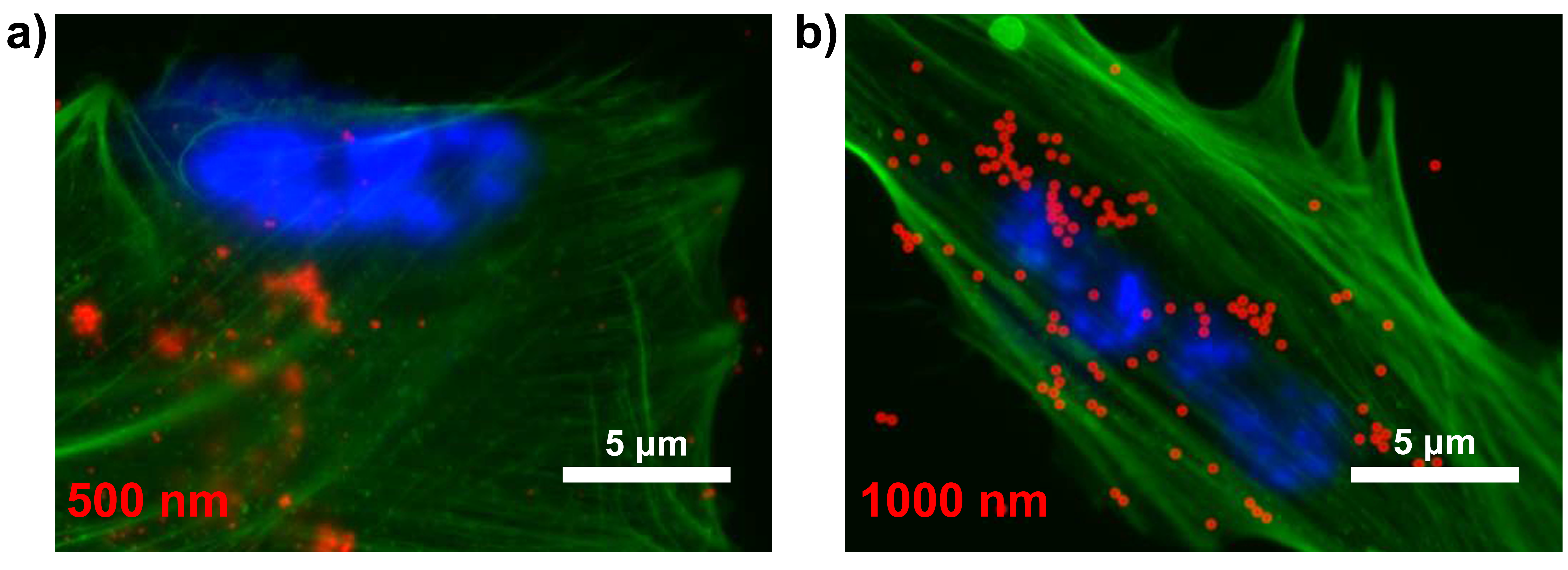


**Figure S5.** Senescent cells were incubated with 25 µg/mL of SiNPs for 80 minutes and imaged at 24 h using a fluorescence microscope. Staining with Hoechst 33342 (blue) and phalloidin-488 (green) shows the nucleus and cytoskeleton, respectively, in relation to internalized NPs (red). Images were taken using a fluorescence microscope. **a-b)** SiNP sizes of 500 and 1000 nm are indicated in the panels.
